# Exercise capacity and body mass index - important predictors of change in resting heart rate

**DOI:** 10.1186/s12872-019-01286-2

**Published:** 2019-12-21

**Authors:** Michal Ehrenwald, Asaf Wasserman, Shani Shenhar-Tsarfaty, David Zeltser, Limor Friedensohn, Itzhak Shapira, Shlomo Berliner, Ori Rogowski

**Affiliations:** Department of Internal Medicine “C”, “D” “E”, Tel Aviv Sourasky Medical Center, Affiliated to the Faculty of Medicine, The Tel Aviv University, 6 Weizmann Street, 64239 Tel Aviv, Israel

**Keywords:** Heart rate, Risk factors, Prevention, Exercise capacity, Body mass index

## Abstract

**Background:**

Resting heart rate (RHR) is an obtainable, inexpensive, non-invasive test, readily available on any medical document. RHR has been established as a risk factor for cardiovascular morbidity, is related to other cardiovascular risk factors, and may possibly predict them. Change in RHR over time (∆RHR) has been found to be a potential predictor of mortality.

**Methods:**

In this prospective study, RHR and ∆RHR were evaluated at baseline and over a period of 2.9 years during routine check-ups in 6683 subjects without known cardiovascular disease from the TAMCIS: Tel-Aviv Medical Center Inflammation Survey. Multiple linear regression analysis with three models was used to examine ∆RHR. The first model accounted for possible confounders by adjusting for age, sex and body mass index (BMI). The 2nd model included smoking status, baseline RHR, diastolic blood pressure (BP), dyslipidemia, high-density lipoprotein (HDL) and metabolic equivalents of task (MET), and in the last model the change in MET and change in BMI were added.

**Results:**

RHR decreased with age, even after adjustment for sex, BMI and MET. The mean change in RHR was − 1.1 beats/min between two consecutive visits, in both men and women. This ∆RHR was strongly correlated with baseline RHR, age, initial MET, and change occurring in MET and BMI (*P* < 0.001).

**Conclusions:**

Our results highlight the need for examining individual patients’ ∆RHR. Reinforcing that a positive ∆RHR is an indicator of poor adherence to a healthy lifestyle.

## Background

### Resting heart rate

Resting heart rate (RHR) is an obtainable, inexpensive, non-invasive test. It is quick, painless, and requires no additional equipment. It is readily available on any chart or medical document.

RHR has been established as a risk factor for cardiovascular morbidity, is related to other cardiovascular risk factors, and may possibly predict them [1–4].

RHR is associated with metabolic disorders, and is elevated in individuals with increased glucose levels, triglycerides, cholesterol levels and body mass index (BMI). RHR is also elevated in individuals who fulfill the standard criteria for metabolic syndrome (MetS), suggesting possible shared pathophysiological mechanisms for both RHR and the MetS [1–3].

RHR appears to be an independent risk factor for heart failure [5, 6]. Some studies have shown RHR to be predictive of all-death mortality and some link RHR to malignancies [7–9].

Many factors affect RHR. Some are non-modifiable determinants such as age, sex, height and race. Others are physiological factors such as influence of the circadian cycle, posture and blood pressure, or lifestyle factors like smoking, alcohol, and mental stress. Physical fitness also affects RHR, and may be expressed by the metabolic equivalent of the task (MET), which is commonly used to express the oxygen requirement of the work rate during a stress test, and demonstrates levels of fitness [1, 10–15]..

### Change in resting heart rate over time

Some studies have examined the effect of change in RHR over time by measuring RHR at baseline and after a period of time (∆RHR). Most of these studies, including large, recent studies, examined the effect of change in heart rate over time in populations on morbidity and mortality, and it has been found to be a potential predictor of both.

Floyd et al. examined 1991 older subjects without known cardiovascular disease and found that 262 subjects had an incident MI event (13%) and 1326 died (67%) during 12 years of median follow-up, concluding that increase in mean RHR and variation in RHR over a period of several years represents a potential predictor of long-term mortality among older persons free of cardiovascular disease [16]. Jiang et al. performed a large cross-sectional and longitudinal study which found that RHR is an independent risk factor for existing metabolic syndrome (MetS) and a predictor for future incidence of MetS, supporting the results of previous studies [3, 13, 17].

Fewer studies have examined the characteristics and risk factors of individual patients, in relation to the change in their RHR (∆RHR) over the years. The HARVEST study found ∆RHR to be an independent predictor of the development of hypertension and of weight gain in young persons screened for stage 1 hypertension [18, 19]. Jouven et al., examined middle-aged Frenchmen employed by the Paris Civil Service between 1967 and 1972 and found ∆RHR to be related to age, tobacco consumption, current sport activity, diabetes mellitus, and blood pressure [20].

In light of evidence that elevated resting heart rate is a risk factor for MetS, heart failure, cardiovascular morbidity and possibly over-all mortality [1–9], the goal of our study is to further examine change in RHR over time in individuals and to determine the factors which affect this change in apparently healthy individuals.

## Methods

### Study design

The study was reviewed and approved by the Tel-Aviv medical center institutional Helsinki Committee (chairperson: Marcel Topilsky and Shmuel Kivity, numbers: 0491–17 and 02–049, at Jan 2002). The data used in this study was collected as part of the “TAMCIS: Tel Aviv Medical Center Inflammation Survey”. Study participants (*n* = 19,385 individuals) were apparently healthy, employed individuals attending a center for periodic health examinations, for a routine health examination during the years 2002–2014 and who gave their written informed consent for participation according to the instructions of the local ethics committee. The routine annual checkups included a physician’s interview and examination, blood and urine tests, and an exercise stress test. Resting heart rate was measured following at least 10 min rest and before the exercise test using electrocardiography ECG, Quinton® Q-Stress (Cardiac Science, Bothell, WA, USA). MET was evaluated using the Quinton Q-stress (Cardiac Science).

Participants were recruited individually by an interviewer while waiting their turn for the clinical examination. They represent 91.6% of the examinees during this period. We systematically checked for nonresponse bias and found that non-participants did not differ from participants on any of the socio-demographic or biomedical variables (see supplementary material for details). Dyslipidemia was defined as serum triglycerides (TG) > 150 mg/dl or use of lipid lowering medications and Low HDL- 40 mg/dl for men and 50 mg/dl for women or use of lipid lowering medications.

Exclusion criteria included the use drugs which may alter heart rate: Use of beta blockers (*n* = 963, 5%), calcium-channel blockers (*n* = 473, 2.4%), antiarrhythmic agents (*n* = 34, 0.17%) or digoxin (*n* = 4, 0.02%). Following this exclusion our cohort for baseline characterization included 18,083 subjects (11,394, 63% men and 6689, 37% women).

Of this baseline cohort, 7735 (42%) subjects arrived for a second routine check-up until May 2014. We further excluded any subjects who changed their smoking status between check-ups (327 subjects who stopped smoking and 233 subjects who started smoking) and the top and bottom 0.3% of ∆RHR. Therefore, our cohort for heart rate change between visits included 6683 subjects (4569, 68.4% men and 2114, 31.6% women).

### Statistical analysis

All data was summarized and displayed as mean ± standard deviation (SD) for the continuous variables and as number of patients plus the percentage in each group for categorical variables. For all categorical variables, the Chi-Square statistic was used to assess the statistical significance between sexes. All above analyses were considered significant at *p* < 0.05 (two-tailed).

Multiple linear regression analyses were used to test heart rate increase over time (∆RHR = RHR at follow-up visit minus RHR at baseline). Three models were examined. The first model accounted for the possible confounders by adjusting for age, sex and BMI. In the 2nd model we added the smoking status, baseline RHR, diastolic blood pressure (BP), dyslipidemia, high-density lipoprotein (HDL-c) and MET (metabolic equivalents) variable, and in the last model, the change in MET and change in BMI were entered into the model. General linear model for heart rate change (Fig. 3) regression included age, sex, basal heart rate, change in MET by quartiles and decrease or increase BMI.

Diabetes Mellitus (DM), hypertension and MetS were defined using IDF and WHO criteria [21, 22].

## Results

The mean change of RHR was − 1.1 beats/min between two consecutive visits for a routine health examination (mean 2.9 ± 1.7 years between visits). As expected from the literature, women presented higher RHR than men (73.0 vs. 69.8 beats/min, *p* < 0.001), lower BMI and blood pressure, lower MET and improved lipid profile (all *p* < 0.001), but the ∆RHR was similar between sexes (*p* = 0.925).

**Fig. 1:**
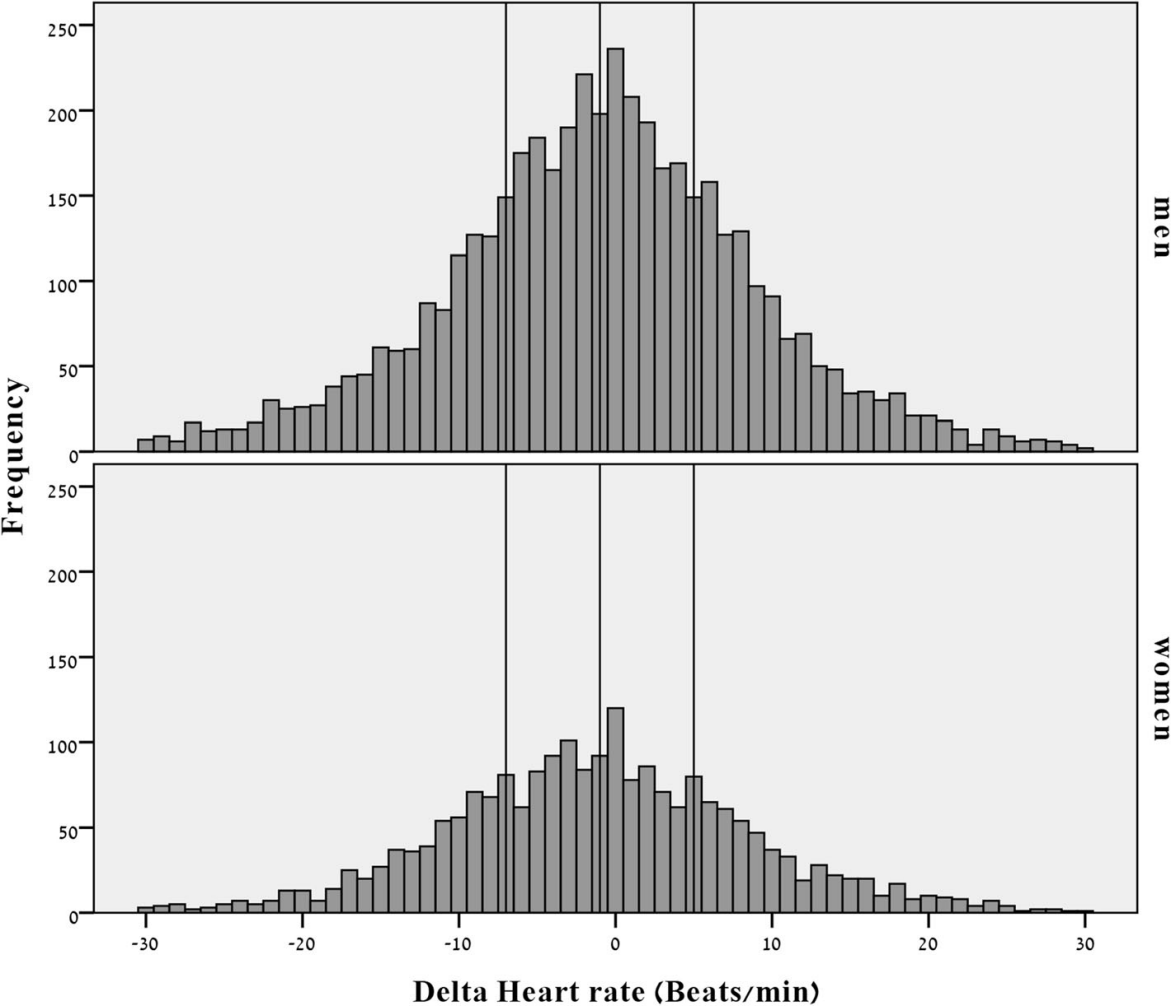
Distribution of ∆RHR by sex (x reference line = − 7, − 1 and 5 representing the ∆RHR quartiles)

Population characteristics presented in Table 1 and the distribution of ∆RHR is shown in Fig. 1 for men and women, with x-axis reference lines dividing the cohort to quartiles of ∆RHR, so that patients in the first two quartiles (left side of the distribution) had lower RHR at the follow-up visit compared to the baseline visit (their RHR decreased with age) while the RHR of those on the right side of the distribution was higher at the baseline measurement than on the follow-up measurement (increased over time).

**Table 1:** Population characteristics

Variable	Gender		
	Men	Women	*P* value
N	11,394	6689	NA
Age, years	43.2 (10.8)	44.1 (10.5)	< 0.001
BMI, kg/m^2^	23.4 (3.4)	20.5 (3.8)	< 0.001
Current smokers, %	16.4	18.3	0.001
Basal heart rate, beats/min	69.8 (12.0)	73.0 (11.4)	< 0.001
Delta heart rate, beats^a^	−1.11 (10.4)	− 1.13 (10.2)	0.925
Systolic BP, mmHg	123.8 (14.1)	116.3 (15.4)	< 0.001
Diastolic BP, mmHg	78.1 (8.3)	73.7 (8.2)	< 0.001
DM, %	3.1	2.7	0.169
Hypertension, %	7.6	5.4	< 0.001
Dyslipidemia, %	15.6	14.3	0.017
MET	12.9 (3.1)	10.3 (2.5)	< 0.001
Sport intensity, h/week	3.2 (15.5)	2.5 (11.3)	< 0.001
HDL- c, mg/dl	50.0 (10.5)	64.1 (15.2)	< 0.001
LDL- c, mg/dl	120.1 (31.2)	116.5 (32.4)	< 0.001
Triglycerides, mg/dl	126.0 (79.5)	100.7 (54.8)	< 0.001

Continuous variables are presented as mean (SD). *BMI* Body mass index. *BP* Blood pressure. *DM* Diabetes Mellitus. *HDL-c* High density lipoprotein cholesterol. *LDL-c* Low density lipoprotein cholesterol. *MET* Metabolic equivalents of task. *Sport intensity* frequency of sport activity per week X length of sport activity. ^a^ Delta HR present the calculated difference between RHR measured at the 2nd visit minus the RHR measured during the first visit

RHR was very weakly correlated with age and BMI (r = − 0.06, r = 0.07 *p* < 0.001) but moderately correlated with MET (r = − 0.39, *p* < 0.001). Controlling for MET revealed stronger negative correlation between RHR and age (r = − 0.205, *p* < 0.001). These results remain significant when we split the analysis by sex.

**Fig. 2:**
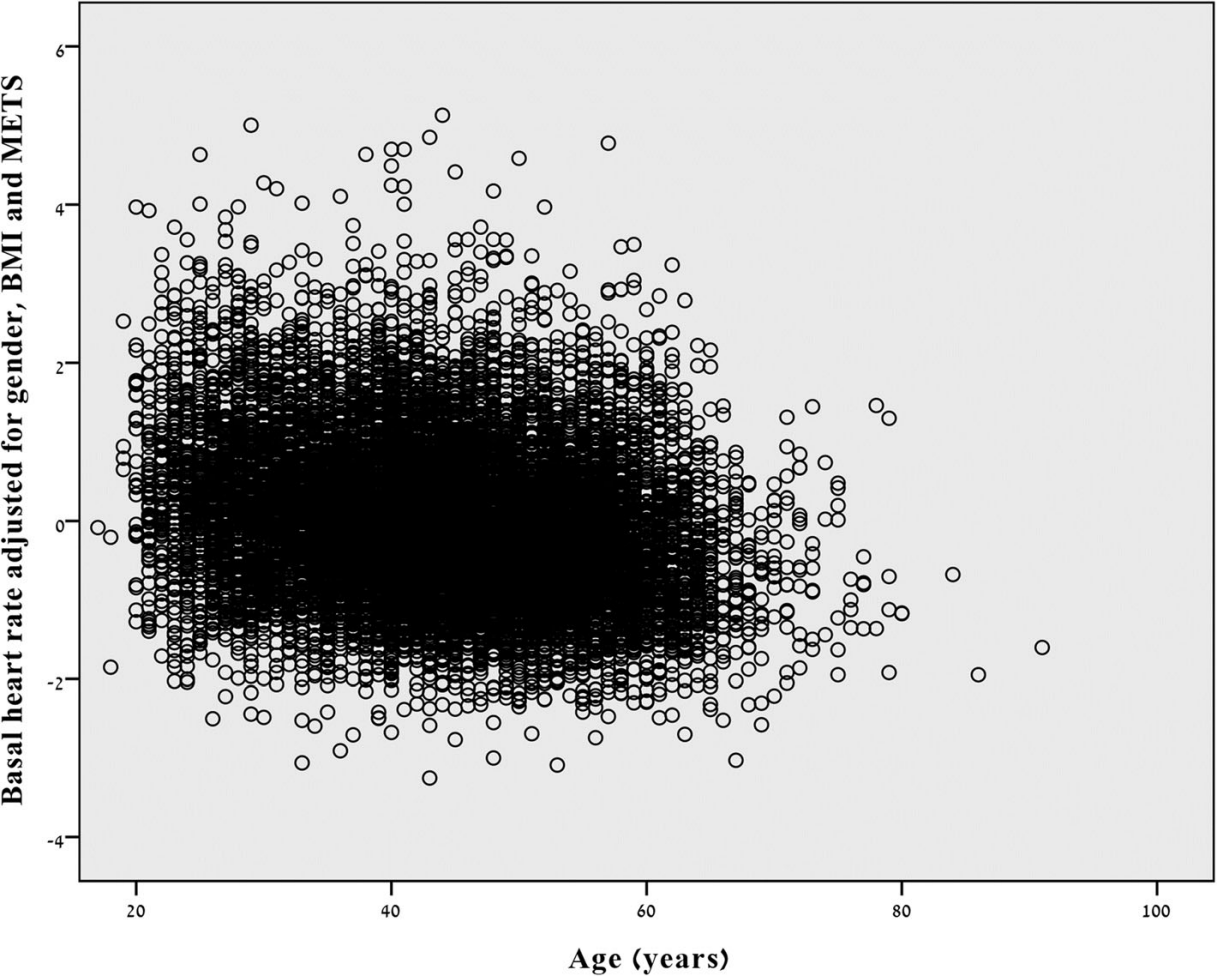
Resting heart rate decreases with age even after adjustment for sex, BMI and MET

Next we adjusted RHR for sex, BMI, and MET and plotted the residuals against age (r = − 0.221, *p* < 0.001), meaning that even after controlling for these 3 confounders, RHR decreased with age (Fig. 2).

### Determinants of increase in resting heart rate

In order to search for possible determinants of increase in annual RHR, we divided subjects into quartiles of ∆RHR and compared their characteristics (See Fig. 1 for distribution). Subjects with increased ∆RHR (increased change between their baseline measurement and follow-up measurement), presented with low baseline RHR and diastolic blood pressure, and a higher frequency of smokers, and elevated MET (p for trend *p* < 0.05, Table 2). Follow up time differed significantly between quartile groups, but the group with lowest ∆RHR had similar follow up time compared to highest ∆RHR group (post hoc *p* value =0.384).

**Table 2:** Population characteristics according to ∆RHR quartiles

Variable	Delta Heart rate					
	1st quartile < (−7)	2nd quartile (− 7) – (−1)	3rd Quartile (−1)- (+ 5)	4th Quartile > (+ 5)	*p* value	P for trend
n	1827	1647	1618	1591	NA	NA
Age, years	44.0 (10.0)	45.4 (9.8)	45.3 (9.9)	44.1 (10.2)	< 0.001	0.616
Gender, male %	66.6	68.8	69.3	69.1	0.272	0.100
BMI, kg/m^2^	22.5 (3.8)	22.7 (3.7)	22.6 (3.7)	22.6 (3.7)	0.684	0.818
Current smokers, %	11.9	12.6	12.9	14.4	0.188	0.038
Basal heart rate, beats/min	78.1 (11.1)	69.8 (10.2)	67.7 (10.2)	65.7 (9.9)	< 0.001	< 0.001
Systolic BP, mmHg	121.6 (14.9)	121.8 (14.7)	121.7 (14.7)	121.3 (14.4)	0.473	0.434
Diastolic BP, mmHg	77.1 (8.5)	76.7 (8.2)	76.6 (8.2)	76.4 (8.1)	0.082	0.013
DM, %	1.8	3.3	3.2	2.3	0.020	0.336
Hypertension, %	7.0	7.3	6.6	6.2	0.594	0.263
Dyslipidemia	15.3	18.5	15.5	17.1	0.044	0.521
MET	11.6 (3.2)	12.3 (3.2)	12.4 (3.3)	12.5 (3.3)	< 0.001	< 0.001
Sport intensity, h/week	2.2 (2.6)	2.3 (2.9)	2.3 (2.7)	2.3 (2.6)	0.131	0.088
HDL- c, mg/dl	55.8 (13.7)	54.8 (14.0)	54.5 (13.6)	54.5 (13.6)	0.02	0.005
LDL- c, mg/dl	123.6 (32.2)	121.7 (31.8)	120.8 (31.2)	121.8 (31.5)	0.087	0.072
Triglycerides, mg/dl	118.5 (74.3)	120.4 (83.3)	116.5 (65.9)	116.3 (66.6)	0.202	0.215
Follow up time, years	2.55 (1.5)	2.48 (1.5)	2.46 (1.4)	2.64 (1.55)	< 0.001	0.120

Continuous variables are presented as mean (SD). *BMI* Body mass index. *BP* Blood pressure. *DM* Diabetes mellitus. *HDL-c* High density lipoprotein cholesterol. *LDL-c* Low density lipoprotein cholesterol. *MET* Metabolic equivalents of task. *Sport intensity* frequency of sport activity per week X length of sport activity

Of special interest was the group of patients with diabetes mellitus (DM); as expected, we found the DM patients had higher RHR compared to DM-free patients (73.5 ± 12.5 vs. 70.3 ± 11.8, *p* < 0.001). One hundred thirty three patients became diabetic during follow-up period. This group had RHR relatively high already during baseline visit (*p* < 0.001) and their ∆RHR remain similar to the group of DM-free patients in both visits (*p* = 0.387).

To assess which variables best explain the variability in heart rate increase, we performed linear regressions (Table 3). Analysis confirmed that age, female sex, baseline RHR, HDL-c and MET have a beneficial effect on heart rate change (decreased ∆RHR), while increasing body weight (expressed as ∆BMI) and presence of dyslipidemia have an adverse effect (increased RHR).

**Table 3:** Linear regression for delta heart rate

	Model 1 (R^2^ = 0.001)			Model 2 (R^2^ = 0.219)			Model 3 (R^2^ = 0.292)		
	β	SE	p	β	SE	p	β	SE	p
Age	0.024	0.016	0.153	−0.040	0.016	0.020	−0.095	0.016	< 0.001
Gender	−0.033	0.362	0.067	0.047	0.396	0.015	−0.048	0.403	0.015
BMI, kg/m^2^	− 0.019	0.046	0.297	0.027	0.044	0.115	−0.004	0.042	0.799
Smoker (y/n)				−0.005	0.417	0.712	−0.011	0.397	0.428
Basal heart rate, beats/min				−0.498	0.014	< 0.001	−0.528	0.013	< 0.001
Diastolic BP, mmHg				0.025	0.019	0.122	0.026	0.018	0.099
Dyslipidemia, (y/n)				0.040	0.391	0.009	0.031	0.373	0.034
HDL-c, mg/dl				−0.044	0.012	0.013	−0.039	0.012	0.021
MET				−0.070	0.56	< 0.001	−0.256	0.061	< 0.001
Delta BMI							0.062	0.074	< 0.001
Delta MET							−0.296	0.065	< 0.001
F change, *p* value	0.172			146.953, *p* < 0.001			187.972, *p* < 0.001		

*BMI* Body mass index. *BP* Blood pressure. *HDL-c* High density lipoprotein cholesterol. *LDL-c* Low density lipoprotein cholesterol. *MET* Metabolic equivalents of task

**Fig. 3:**
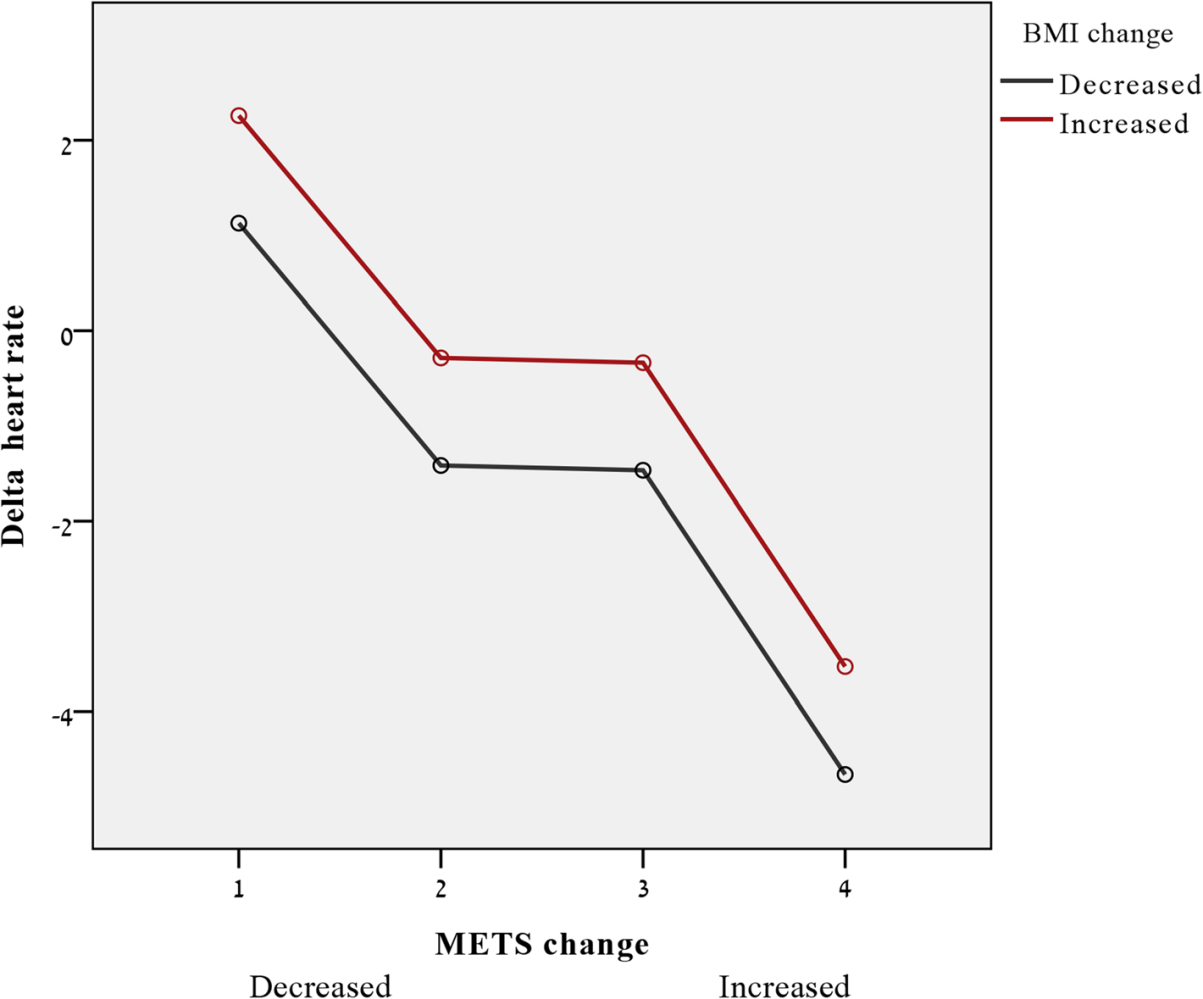
Plot of estimated effect on delta heart rate of both MET change (x axis, 1 = lowest, 4 highest MET change) and change of BMI (color coded, decreased = black, increased = red). Y axes indicate the estimated effect on delta heart rate (based on general linear regression model following adjustment to age, sex and basal heart rate

Figure 3 presents the inverse trends of MET and BMI with ∆RHR, demonstrating that individuals who increased exercise capacity on follow-up visit (x = 3 and 4) showed decreased heart rate compared to those with decreased exercise capacity (x = 1) who showed increased heart rate. Reduction of BMI also resulted in decreased heart rate, and vice versa.

## Discussion

In this cohort study of healthy, employed adults without known cardiovascular disease, we found that RHR decreased with age, even after adjustment for sex, BMI and MET. We found that RHR values tend to decrease with aging (mean change in RHR was − 1.1 beats/min per 2.9 years of follow-up), so that RHR increases are the exception, and chose to focus on the correlations for this change, emphasizing the importance of recognizing increased annual heart rate as another risk factor for cardiovascular disease and mortality. We showed that age, female sex, baseline RHR, HDL-c and MET have a beneficial effect on heart rate change (decreased RHR), while increasing body weight (expressed as ∆BMI) and presence of dyslipidemia have an adverse effect (increased RHR).

Elevated RHR has been established as a risk factor for cardiovascular morbidity and mortality. Some studies have shown RHR to be predictive of all-death mortality and some link RHR to malignancies [7–9].

Our study found that RHR decreased with age. The effect of age on RHR is less well-established than that of other non-modifiable determinants [23–25]. In some studies there appears to be a decrease in RHR with age [12, 26–29]. Some show no change in RHR or a decrease followed by a plateau [12, 29–32]. Others found a decrease in RHR in women but not in men [8, 30]. Palatini et al. found an increase in RHR with age, in patients with isolated systolic hypertension [33].

We found annual increased heart rate to be correlated with a decrease in MET. The metabolic equivalent of the task (MET) is commonly used to express the oxygen requirement of the work rate during a stress test, and demonstrates levels of fitness. Decreased MET, therefore, implies less efficient heart function and worse cardiovascular fitness.

Generally, cardiac output and heart rate decrease with age [34, 35].), therefore, individuals with an increasing trend of RHR should be encouraged to choose and maintain a healthy lifestyle in order to decrease cardiovascular risks. Nevertheless, the clinical significance of individual ∆RHR remains to be examined in larger-scale cohorts.

Furthermore, our findings highlight the importance of fitness and heart rate tracking. The development in personal digital devices has made heart rate information as important and easily access as ever, thus, provides a golden opportunity to identify use delta heart rate as an early marker of disease prevention. However, currently there are no general recommendations to guide the general public in this area [36].

Heart rate is controlled by the autonomic nerves, with sympathetic stimulation increasing the rate and parasympathetic stimulation decreasing it. Athletes have lower RHR compared with the general population and training results in reduced RHR. This is explained by changes the sympathovagal balance of the sinus node. In response to regular exercise changes occur in the cardiovascular system, such as increased contraction due to cardiac muscle fibers hypertrophy and increased muscle mass of the ventricles. Oxygen and nutrient delivery to muscles is improved by enhanced capillary capacity for blood flow, which causes a decrease in total peripheral resistance [37].

The importance of fitness to health is well-known and studied, and there is an inverse relationship between fitness and mortality. Kokkinos et al. examined MET in 18,102 men who were followed for a median of 10.8 years, and found that for each additional in exercise capacity of 1-MET, mortality risk was reduced by 12% [38].

We also found annual increased heart rate to be correlated with increase in BMI. The HARVEST study found ∆RHR to be an independent predictor of the development of weight gain in young persons screened for stage 1 hypertension, and several studies link increased RHR and metabolic syndrome.

High BMI is a well-known cardiovascular risk factor, even in metabolically-healthy overweight people [39]. Interestingly, the increase in BMI seen in our study population remained in the normal range of BMI and our cohort was not over-weight or obese. Therefore, increase in RHR was affected by increase in BMI even in individuals with normal BMI.

This study analyzed the correlations of annual ∆RHR; it does, however, have limitations. While initial study population included 18,083 subjects after applying exclusion criteria (see “Methods”), at the second time point we were able to follow-up on only 7735 (42%) subjects, who actually arrived at our center for annual routine checkups. We systematically checked for non-response bias and found that non-participants did not differ from participants on any of the socio-demographic or bio-medical variables. Furthermore, a high rate of participation was observed (91.6% of those who arrived at the center and were asked to participate). Another limitation is that our cohort consisted of participants in a health screening program and is not a population-based sample. However, the mean BMI in our study is very similar to that published by the National Health Survey from Israel [40]. Second, mean follow-up was 3 years, which is a relatively short time period for the investigation of RHR trajectories. Nevertheless, we were able to show a significant link between the biomarker studied and RHR trends in this cohort of apparently healthy individuals. We expect that the observed trend will increase with time unless modification in life style will be done. Another possible limitation is that the use of baseline RHR measurements introduced potential bias with respect to regression to the mean. Suggesting that individuals with extreme RHR on its basal measurement but closer to the mean on its second measurement [41]. The very large spread in the change in RHR (Fig. 1) are most likely not due to biological factors, but other factors such as measurement error, change in measuring circumstances, regression towards the mean, and others. Last but not list, it was previously shown that among employees, white collar workers (professional, managerial, clerical) had slightly higher resting heart rates than blue collar workers. This suggests a possible effect of greater physical fitness among blue collar workers [42]. However, in our study we did not collect data regarding type and amount of physical activity during work hours.

## Conclusions

Our results confirm that RHR decreases with age and strengthens the need for identifying patients with annual RHR increase as a population at risk. Determinants of increased annual RHR over time and its consequences include initial lower exercise capacity, dyslipidemia, increase of BMI or decrease in MET.

Our findings may be useful in identifying easily, and without any additional cost or time, asymptomatic individuals at risk, who could benefit from primary prevention (lifestyle changes or medication) in order to reduce cardiovascular risk.

## Data Availability

The datasets used and/or analysed during the current study are available from the corresponding author on reasonable request.

## Abbreviations

∆RHR: Delta resting heart rate, representing change in resting heart rate over a period of time
BMI: Body mass index
BP: Blood pressure
DM: Diabetes mellitus
HDL-c: High density lipoprotein cholesterol
LDL-c: Low density lipoprotein cholesterol
MET: Metabolic equivalents of task
MetS: Metabolic syndrome
MI: Myocardial infarction
RHR: Resting heart rate
